# Oxidative Stress and Antioxidant Defense Mechanisms in Acute Ischemic Stroke Patients with Concurrent COVID-19 Infection

**DOI:** 10.3390/ijms242316790

**Published:** 2023-11-27

**Authors:** Elena Anca Pinoșanu, Roxana Surugiu, Emilia Burada, Denisa Pîrșcoveanu, Camelia Elena Stănciulescu, Raluca Elena Sandu, Cătălina Pisoschi, Carmen Valeria Albu

**Affiliations:** 1 Department of Neurology, University of Medicine and Pharmacy of Craiova, St. Petru Rares, No. 2-4, 200433 Craiova, Romania; elenapinosanu@yahoo.com (E.A.P.); denisa.pirscoveanu@umfcv.ro (D.P.); carmen.albu@umfcv.ro (C.V.A.); 2 Doctoral School, University of Medicine and Pharmacy of Craiova, St. Petru Rares, No. 2-4, 200433 Craiova, Romania; 3 Department of Biochemistry, University of Medicine and Pharmacy of Craiova, St. Petru Rares, No. 2-4, 200433 Craiova, Romania; roxana.surugiu@umfcv.ro (R.S.); camelia.stanciulescu@umfcv.ro (C.E.S.); 4 Department of Physiology, University of Medicine and Pharmacy of Craiova, St. Petru Rares, No. 2-4, 200433 Craiova, Romania; emilia.burada@umfcv.ro

**Keywords:** stroke, COVID-19, oxidative stress, inflammation, SARS-CoV-2

## Abstract

Stroke remains a debilitating cerebrovascular condition associated with oxidative stress, while COVID-19 has emerged as a global health crisis with multifaceted systemic implications. This study investigates the hypothesis that patients experiencing acute ischemic stroke alongside COVID-19 exhibit elevated oxidative stress markers and altered antioxidant defense mechanisms compared to those with acute ischemic stroke. We conducted a single-center prospective cross-sectional study to investigate oxidative stress balance through oxidative damage markers: TBARS (thiobarbituric acid reactive substances level) and PCARB (protein carbonyls); antioxidant defense mechanisms: TAC (total antioxidant capacity), GPx (glutathione peroxidase), GSH (reduced glutathione), CAT (catalase), and SOD (superoxide dismutase); as well as inflammatory response markers: NLR (neutrophil-to-lymphocyte ratio), CRP (C-reactive protein), and ESR (erythrocyte sedimentation rate). Statistical analyses and correlation models were employed to elucidate potential associations and predictive factors. Our results revealed increased oxidative stress, predominantly indicated by elevated levels of TBARS in individuals experiencing ischemic stroke alongside a concurrent COVID-19 infection (*p* < 0.0001). The Stroke-COVID group displayed notably elevated levels of GSH (*p* = 0.0139 *), GPx (*p* < 0.0001 ****), SOD (*p* = 0.0363 *), and CAT (*p* = 0.0237 *) activities. Multivariate analysis found a significant association for TBARS (*p* < 0.0001 ****), PCARB (*p =* 0.0259 *), and GPx activity (*p* < 0.0001 ****), together with NLR (*p* = 0.0220 *) and CRP (*p* = 0.0008 ***). Notably, the interplay between stroke and COVID-19 infection appears to amplify oxidative damage, potentially contributing to exacerbated neurological deficits and poorer outcomes. This study highlights the intricate relationship between oxidative stress, inflammation, and concurrent health conditions. Understanding these interactions may open avenues for novel therapeutic strategies aimed at ameliorating oxidative damage in patients with acute ischemic stroke and COVID-19, ultimately improving their prognosis and quality of life.

## 1. Introduction

According to the recently published Global Burden of Disease (GBD) 2019 report, stroke remains a prominent global health issue, ranking as the second most prevalent cause of mortality and the third leading cause of both mortality and disability when assessed through disability-adjusted life-years lost (DALYs) [1], with ischemic strokes accounting for more than 62% of all reported stroke incidents [2].

Simultaneously, the COVID-19 pandemic has emerged as an unprecedented public health crisis [3,4] affecting millions [5]. While both stroke and COVID-19 represent distinct challenges, recent research has illuminated their intricate connection, revealing a complex interplay between the two conditions [6,7,8].

Oxidative stress refers to an imbalance between the production of reactive oxygen species (ROS) and the ability of biological systems to effectively detoxify or repair any resulting damage [9]. Oxidative stress is thought to play a pivotal role in the initiation and progression of strokes through a complex network of interconnected mechanisms [10]. In ischemic stroke, increased ROS production and/or impaired ROS degradation [11] serves as a significant catalyst and propagator of neuronal dysfunction and mortality [12], and is a crucial detrimental factor in cerebral ischemia [13]. On the other hand, the role of oxidative stress and hyper-inflammatory response in SARS-CoV-2 infection seems to be a complex phenomenon in the pathogenesis of the disease [14].

The assay for thiobarbituric acid reactive substances (TBARS) has been extensively employed as a universal metric for assessing lipid peroxidation in biological fluids. Widely recognized as a reliable indicator, it is frequently utilized to gauge the extent of oxidative stress within a biological sample [15]. Another good indicator of the oxidative damage is the evaluation of protein ‘carbonylation’, a significant indicator of oxidative damage to proteins that involves the introduction of carbonyl groups, such as aldehyde, ketone, and lactam, into the side chains of amino acids [16].

The detrimental effects are mitigated by the presence of antioxidant systems, composed of molecules with the ability to neutralize reactive oxygen species (ROS), thereby averting the initiation of oxidative damage [17]. Among these, glutathione peroxidase (GPx) stands out as an enzyme endowed with antioxidant properties, playing a pivotal role at the cellular level in alleviating oxidative stress. Its function involves catalyzing the reduction of organic hydroperoxides, utilizing the antioxidant glutathione (GSH) [17,18]. In this process, GPx actively participates in transforming reactive oxygen species (ROS) into benign compounds, namely oxygen and water, thereby contributing significantly to the cellular defense against oxidative damage [19]. Other key enzymes include superoxide dismutase (SOD), a crucial antioxidant enzyme that facilitates the transformation of superoxide into hydrogen peroxide and oxygen, impacting immunological responses [20], and catalase, heme enzyme that plays a crucial role as integral members of the antioxidant defense system found in the cells of virtually all aerobic organisms [21].

The hypothesis of this study is that patients with acute ischemic stroke, while concurrently infected with COVID-19, will display heightened oxidative stress markers and altered antioxidant defense mechanisms compared to patients with acute ischemic stroke alone. Our work investigates the balance of oxidative stress by analyzing a range of indicators: TBARS and PCARB for oxidative damage, and a comprehensive set of antioxidant defense mechanisms, including total antioxidant capacity (TAC), GPx, GSH, CAT, and SOD. We postulate that the combined impact of stroke and COVID-19 infection leads to an intricate interplay between oxidative stress and inflammation, contributing to more severe oxidative damage in these patients.

## 2. Results

### 2.1. Clinical and Demographic Characteristics

The analysis of the control and the COVID groups demonstrates significant differences in various important variables. The average age of individuals in the COVID and control group was similar, with a mean age of 72.87 years (range: 59–89) for the Stroke-COVID group, and an average age of 70 years (range: 42–87) for the control group. The analysis of gender distribution revealed that the COVID group exhibited a significantly greater representation of women (57.5%) in comparison to the control group (38.9%), *p* = 0.0331. Furthermore, there was no statistically significant difference between the two groups in regard to the incidence of hypertension (96% vs. 86.9%), diabetes mellitus (26% vs. 21.7%), and atrial fibrillation (30% vs. 17.3%). It is noteworthy that the prevalence of dyslipidemia among the risk factors is significantly higher in individuals with COVID-19 (56% vs. 19.5%), with a *p*-value of less than 0.0001. The aforementioned findings are presented in Table 1.

### 2.2. Evaluation of the Oxidative Stress and Inflammatory Response Markers

We focused on two key oxidative damage markers: TBARS (thiobarbituric acid reactive substances level) and PCARB (protein carbonyls), commonly employed biomarkers for lipid peroxidation and protein oxidation, respectively. We assessed the status of antioxidant defense mechanisms by measuring total antioxidant capacity (TAC), as well as the activity of key enzymes including glutathione peroxidase (GPx), superoxide dismutase (SOD), and catalase (CAT), along with the concentration of reduced glutathione (GSH).

Furthermore, the inflammatory response was assessed through the measurement of several key markers: neutrophil-to-lymphocyte ratio (NLR), C-reactive protein (CRP), and erythrocyte sedimentation rate (ESR) (Table 2).

Our findings identified heightened oxidative stress primarily in the form of elevated TBARS levels in patients with ischemic stroke and concurrent COVID-19 infection (*p* < 0.0001). While protein oxidation (PCARB) showed a subtle increase in the Stroke-COVID group, it did not reach statistical significance, suggesting that lipid peroxidation plays a more prominent role in this dual pathology.

While TAC remained relatively stable between the two groups, the Stroke-COVID group exhibited significantly higher levels of GSH, GPx, SOD, and CAT activities. These findings suggest an enhanced antioxidant defense mechanism in response to the combined stressors of stroke and COVID-19.

The assessment of the inflammatory response revealed a heightened and significant elevation in all evaluated key inflammatory markers (NLR: *p* = 0.0211; CRP: *p* < 0.0001; ESR: *p* = 0.0287) in patients with acute ischemic stroke and comorbid COVID-19 infection.

We conducted a comprehensive multivariate analysis to examine the intricate relationships among oxidative stress markers, antioxidant defense mechanisms, and inflammatory response parameters in patients with acute ischemic stroke and concurrent COVID-19 infection. The results revealed statistically significant association for TBARS (*p* < 0.0001 ****), PCARB (*p* = 0.0259 *), and GPx activity (*p* < 0.0001 ****), together with NLR (*p* = 0.0220 *) and CRP (*p* = 0.0008 ***), highlighting an augmented inflammatory response in this cohort.

### 2.3. Receiver Operating Characteristic Curve (ROC) Analysis

**Figure 1:**
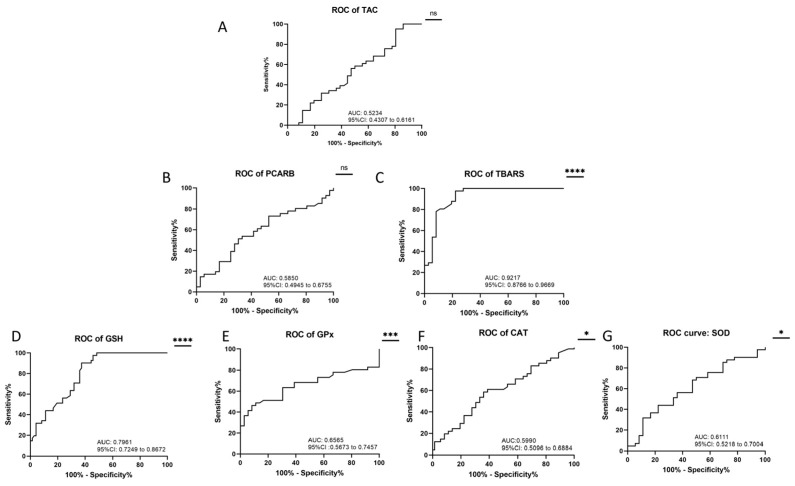
ROC analysis of (**A**) TAC (AUC: 0.5234; 95% CI: 0.4307 to 0.6161, *p* = 0.6173); (**B**) PCARB (AUC: 0.5850; 95% CI: 0.4945 to 0.6755, *p* = 0.0691); (**C**) TBARS (AUC: 0.9217; 95% CI: 0.8766 to 0.9669, *p* < 0.0001); (**D**) GSH (AUC: 0.7961; 95% CI: 0.7249 to 0.8672, *p* < 0.0001); (**E**) GPx (AUC: 0.6565; 95% CI: 0.5673 to 0.7457, *p* = 0.0008); (**F**) CAT (AUC: 0.5990; 95% CI: 0.5096 to 0.6884, *p* = 0.0343); (**G**) SOD (AUC: 0.6111; 95% CI: 0.5218 to 0.7004, *p* = 0.0175). * *p* < 0.05; *** *p* < 0.001; **** *p* < 0.0001.

ROC analysis is a valuable statistical tool used to evaluate the ability of a diagnostic test or biomarker to distinguish between two groups; in this case, patients with acute ischemic stroke alone and those with acute ischemic stroke and COVID-19 infection. ROC analysis was performed to determine the sensitivity and specificity of the investigated oxidative stress markers (Figure 1).

We can point out that based on the AUC values of the investigated markers, we identified an excellent discriminatory performance for TBARS (AUC: 0.9217; 95% CI: 0.8766 to 0.9669, *p* < 0.0001), a good discriminatory performance for GSH (AUC: 0.7961; 95% CI: 0.7249 to 0.8672, *p* < 0.0001), and a fair discriminatory performance for GPx (AUC: 0.6565; 95% CI: 0.5673 to 0.7457, *p* = 0.0008) and SOD (AUC: 0.6111; 95% CI: 0.5218 to 0.7004, *p* = 0.0175), while the rest exhibited only a limited ability to differentiate between the two groups.

### 2.4. Correlation of Oxidative Stress Markers with the Inflammatory Response

**Figure 2:**
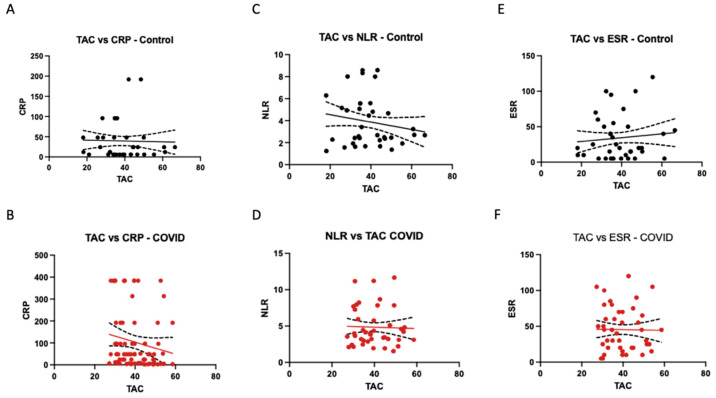
Total antioxidant capacity (TAC) vs. inflammatory markers: C-reactive protein (CRP) in the control (**A**) and Stroke-COVID (**B**) groups; neutrophile-to-lymphocyte ratio (NLR) in the control (**C**) and Stroke-COVID (**D**) groups; erythrocyte sedimentation rate (ESR) in the control (**E**) and Stroke-COVID (**F**) groups.

In this analysis, we explored the relationships between total antioxidant capacity (TAC) and various inflammatory markers, including C-reactive protein (CRP), neutrophil-to-lymphocyte ratio (NLR), and erythrocyte sedimentation rate (ESR) (Figure 2).

Our analysis did not reveal a statistically significant association between TAC and the inflammatory response markers in our study. Further investigations are needed to explore other potential factors contributing to the inflammatory response in these patients.

### 2.5. Correlation of Oxidative Stress Markers with Neurological Scales

**Figure 3:**
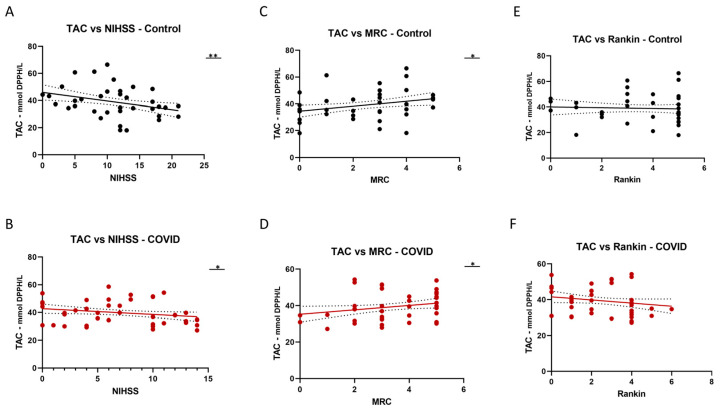
Total antioxidant capacity (TAC) vs. NIHHS (National Institutes of Health Stroke Scale) in the control (**A**) and Stroke-COVID (**B**) groups; total antioxidant capacity (TAC) vs. MRC (Medical Research Council) scale in the control (**C**) and Stroke-COVID (**D**) groups; total antioxidant capacity (TAC) vs. Rankin Scale in the control (**E**) and Stroke-COVID (**F**) groups. * *p* < 0.05; ** *p* < 0.01.

Additionally, we explored the correlation between total antioxidant capacity (TAC) levels and stroke prognostic scales, namely the NIHHS, MRC, and Rankin scales (Figure 3).

We observed a negative correlation between TAC levels and the NIHHS scale in both groups (control group: *p* = 0.0071, Stroke-COVID group: *p* = 0.0415). TAC levels exhibited a positive correlation with the MRC scale in the control group (*p* = 0.0181), but not in the Stroke-COVID group (*p* = 0.0494). However, we noted a negative correlation with the Rankin scale, which did not reach statistical significance.

### 2.6. Correlation of Oxidative Stress Markers and Inflammatory Response with Neurological Scales

Finally, we conducted an analysis of the association between NIHSS neurological severity scales and the investigated markers related to oxidative stress, antioxidant defense, and inflammation using multiple linear regression, as presented in Table 3.

We established a significant association through multiple linear regression between NIHSS and TBARS (*p* = 0.0251), TAC (*p* = 0.0039), CRP (*p* = 0.0478), and the Rankin (*p* < 0.0001), as well as MRC (*p* = 0.0050) scales, thus reinforcing our earlier findings.

## 3. Discussion

Oxidative stress, characterized by an imbalance between the production of ROS and the body’s antioxidant defense mechanisms [9], has emerged as a pivotal player in the pathophysiology of numerous neurological disorders [12,13]. Among these, stroke stands prominently linked with oxidative stress [11] as a leading cause of morbidity and mortality worldwide [1], with its multifaceted etiology and complex evolution challenging both clinicians and researchers alike. More recently, the global healthcare landscape has been further convoluted by the emergence of the novel coronavirus disease 2019 (COVID-19), caused by the severe acute respiratory syndrome coronavirus 2 (SARS-CoV-2) [22]. Numerous studies discuss the importance of oxidative stress in the COVID-19 pathology [14,23]. The interplay between oxidative stress, stroke, and COVID-19 represents a dynamic and evolving area of investigation.

The findings of our study highlight the complex interplay between oxidative stress, antioxidant defense mechanisms, and inflammation in patients presenting with acute ischemic stroke and a concomitant COVID-19 infection. The demographic analysis showcased comparable ages between the Stroke-COVID and control groups, while a higher representation of women and a significant prevalence of dyslipidemia emerged as notable features in the COVID group. Delving into oxidative stress markers, elevated TBARS levels in the Stroke-COVID group underscored the heightened state of cellular damage, complemented by increased antioxidant defense mechanisms, particularly in GSH, GPx, SOD, and CAT activities, compared to the control group. The inflammatory response exhibited significant elevation in NLR, CRP, and ESR levels in the Stroke-COVID group, emphasizing the interconnected nature of these physiological responses. Multivariate analysis reinforced these associations, with TBARS, PCARB, GPx, NLR, and CRP demonstrating statistically significant connections. Several key observations emerge from our analysis.

Firstly, our results underscore the substantial burden of comorbidities in the COVID-19 group; particularly noteworthy is the significantly elevated occurrence of dyslipidemia (*p* < 0.001) among these risk factors, emphasizing the importance of lipid metabolism in the context of COVID-19. Similar findings were previously reported [24,25]. Dyslipidemia was identified by multiple studies as a risk factor related to COVID-19 severity, with different prevalence ranges from 1 to 10% in Asia [26,27,28], to 28% in France [29], and up to 32.5% in the USA [30], indicating the possibility that dyslipidemia may contribute to the severity of COVID-19 infection [31].

In terms of oxidative stress, we observed a marked increase in lipid peroxidation, as indicated by elevated TBARS levels in patients with both ischemic stroke and COVID-19, compared to the control group. This suggests a potentiation effect, where the co-occurrence of these conditions leads to exacerbated oxidative damage [32,33,34,35,36,37,38]. While protein oxidation (PCARB) displayed a subtle elevation, the absence of statistical significance implies that lipid peroxidation plays a more substantial role in this combined pathology. TBARS was previously identified as an elevated marker in COVID-19 patients [39], as well as acute ischemic stroke patients associated with a poorer outcome [40].

Moreover, the enhanced antioxidant defense mechanisms in the Stroke-COVID group, characterized by increased GSH, GPx, SOD, and CAT activity, suggest a concerted effort to counteract the oxidative stress provoked by the dual insult. Previous studies on COVID-19-positive patients reported mixed results, with SOD levels identified as decreased by Liao et al. [41], while other studies reported increased SOD levels, associated with increased CAT and GPx levels [23,42], concurring with our findings.

Our assessment of the inflammatory response unveiled a significant and consistent elevation in key inflammatory markers, including NLR, CRP, and ESR, in the Stroke-COVID group. This heightened inflammatory state suggests a synergistic interaction between ischemic stroke and COVID-19, leading to a robust pro-inflammatory milieu. CRP was previously reported as a robust indicator of disease severity in COVID-19 patients [23], serving as a significant indicator of fatalities in individuals diagnosed with COVID-19 [43].

Remarkably, our analysis of the discriminative abilities of these markers demonstrated excellent performance for TBARS and good performance for GSH, highlighting their potential as diagnostic tools in the context of comorbid ischemic stroke and COVID-19.

It must be emphasized that oxidative stress is inevitably linked to age. The rise of oxidative products and the alterations of the antioxidant status are determined by the imbalance between the rate of ROS formation in the biological systems and the activity of antioxidants in older people. Additionally, other factors, such as drugs and systemic diseases, might influence redox homeostasis. That is why it was difficult to select a group of healthy subjects, homogeneous in age and sex with the stroke groups, having values between the normal range for all the oxidative stress parameters, and we decided to perform a comparison between both stroke groups.

This study’s findings provide a foundation for further research exploring the complex relationships between oxidative stress, inflammation, and stroke, especially within the context of COVID-19. Subsequent investigations are imperative to fully elucidate the prognostic potential inherent in these markers for individuals experiencing concurrent stroke and COVID-19. Moreover, there exists a compelling opportunity for the development of precision therapeutic strategies and the implementation of nuanced, individualized patient care. This study not only contributes valuable insights but also underscores the necessity for continued exploration in this complex and clinically relevant domain.

## 4. Materials and Methods

### 4.1. Ethical Consent

The current investigation received approval (Registration no. 202/19 October 2022) from the Academic and Scientific Ethics and Deontology Committee at the University of Medicine and Pharmacy in Craiova, Romania, in accordance with the guidelines set forth by the European Union (Declaration of Helsinki). Each of the patients provided their signature indicating their consent and agreement to participate in the current study.

### 4.2. Study Design

It was a single-center prospective cross-sectional study, carried in the Neuropsychiatry Hospital of Craiova from October 2022 to February 2023. Patients or their authorized representatives were provided with information regarding the study, and written informed consent was obtained from eligible participants who met the inclusion and exclusion criteria.

### 4.3. Participants

The research team conducted a screening process to determine the eligibility of patients admitted to our hospital who had been diagnosed with acute ischemic stroke for inclusion in the study. The inclusion criteria for this study were as follows: (1) individuals of both genders who are above the age of 18; (2) individuals diagnosed with ischemic stroke; (3) individuals who experienced the onset of symptoms within a period of less than 24 h; (4) individuals with a measurable neurological deficit, as indicated by a National Institutes of Health Stroke Scale (NIHSS) score greater than 3 and less than 22; and (5) individuals who are not eligible for thrombolytic therapy or mechanical thrombectomy. The criteria for exclusion encompassed the following factors: (1) patients with a severe stroke, as indicated by a NIHSS score greater than 22; (2) patients with a hemorrhagic stroke; (3) patients with evidence of other diseases or conditions affecting the central nervous system (CNS), such as demyelinating disorders, brain tumors, previous craniotomy, and severe brain injury; (4) the presence of psychological, pharmacological, or medical factors that may interfere with the collection or interpretation of trial data; and (5) other concomitant infections, except COVID in the Stroke-COVID group.

The identification of acute ischemic stroke was achieved by means of clinical and neurological assessments, as well as cerebral imaging techniques such as computed tomography (CT).

The patients were admitted to the hospital and placed in an observation room pending the administration of a COVID test. For the Stroke-COVID group, we exclusively enrolled patients who tested positive for COVID-19 at the time of their stroke diagnosis. Individuals who tested positive for COVID-19 were assessed by an expert in infectious diseases, in accordance with the prescribed procedure for laboratory testing, thoracic CT scans, and medical intervention. In addition, the specialist facilitated the evaluation of the severity of COVID in accordance with the scale endorsed by the World Health Organization (WHO).

A total of 96 patients, diagnosed with ischemic stroke (mild and severe) between October 2022 and February 2023, were included in this study. The patients were divided in two groups: control (patients with ischemic stroke—N = 46) and COVID (patients with ischemic stroke and SARS-CoV-2 infection—N = 50), and clinical and demographic data were collected. The assessment of lesion location, modified Rankin Scale (mRS), Medical Research Council (MRC), and NIHSS scores was conducted by a specialist who had received appropriate training.

### 4.4. Examination Tools

The current form of the Rankin scale was modified by Charles Warlow and colleagues during the UK-TIA trial in the 1980s [44]. Its reproducibility was initially assessed by van Swieten et al. in 1988 [45] and possesses several notable strengths. Firstly, it encompasses the complete spectrum of functional outcomes, ranging from the absence of symptoms to mortality (Supplementary Table S1). Secondly, the categories employed in the scale are intuitive and readily comprehensible to both medical practitioners and patients. Thirdly, the mRS exhibits concurrent validity, as evidenced by its robust correlation with measures of stroke pathology, such as infarct volumes, and its coherence with other stroke scales [46].

The Medical Research Council (MRC) muscle strength scale is commonly employed in the fields of neurology and rehabilitation for evaluating muscle strength in a variety of medical conditions [47] including stroke [48]. The scale is structured in a numerical sequence ranging from 0 to 5, in which each grade corresponds to a distinct level of muscular strength (Supplementary Table S2) [49].

The National Institutes of Health Stroke Scale (NIHSS) is a validated and widely used standardized instrument for assessing and quantifying the extent of neurological impairments in individuals who have experienced a stroke [50]. It comprises 15 individual items that evaluate various domains of neurological function: the assessment of consciousness, eye movement, visual abilities, facial expressions, muscular strength in the arms and legs, sensory perception, motor coordination, linguistic proficiency, speech capabilities, and the presence of neglect. Each individual item is assigned a score ranging from 0, representing a state of normalcy, to a maximum of 2, 3 or 4, contingent upon the specific item being evaluated (Supplementary Table S3). The NIHSS score in its entirety offers a quantitative assessment of the severity of a stroke, whereby higher scores correspond to more pronounced deficits [51].

### 4.5. Collection and Handling of Samples

The blood samples were obtained in the morning, following a 12 h fasting period by a skilled phlebotomist using EDTA (ethylene-diamine-tetraacetic acid) tubes for collection, in the first 48 h after the stroke diagnosis. The plasma and red blood cell (RBC) fractions were isolated through the process of centrifugation, which involved spinning the sample at a speed of 3600 revolutions per minute (rpm) at a temperature of 4 °C for a duration of 10 min using an Eppendorf refrigerated centrifuge model 5417 R. The separation of sediments from plasma occurred promptly following centrifugation, and subsequent analysis of oxidative stress markers was conducted on the plasma. The plasma samples were stored at a temperature of −80 °C until they were ready for use. The act of repeatedly thawing and freezing samples was prevented.

### 4.6. Assay for Thiobarbituric Acid Reactive Substances (TBARS)

In order to evaluate the levels of lipid peroxidation, we performed a plasma analysis of thiobarbituric acid reactive substances (TBARS) using an UV spectrophotometric method, as described in previous studies [52,53,54,55,56]. The present analysis relies upon the utilization of malondialdehyde (MDA) as a widely employed biomarker for the assessment of oxidative stress. The extent of lipid peroxidation was assessed through the quantification of MDA concentration in deproteinized human plasma.

In brief, a 0.1 mL sample of human plasma was subjected to treatment using a solution consisting of 5% trichloroacetic acid (TCA) and 0.2 M Tris–HCl at a pH of 4.7 (*v*/*v*). Following a 10 min incubation period at ambient temperature, the sample was combined with 1 mL of a solution containing 0.55 M thiobarbituric acid (TBA) in 2 M sodium sulphate. The resulting mixture was subjected to heating at a temperature of 90 °C for a duration of 45 min, after which it was promptly cooled using an ice bath [57]. Following this, the optical density (OD) of the sample was measured at a wavelength of 532 nm utilizing a UV-VIS spectrophotometer (Kruss, Hamburg, Germany). The concentration of TBARS was determined using the molar extinction coefficient of MDA (1.55 × 10^5^ M^−1^ cm^−1^), and the findings are reported in terms of TBARS (µmol/L).

The reagents were purchased from Sigma Aldrich (St. Louis, MO, USA, TCA, TBA, TRIS–HCl) and Merck KGaA (Darmstadt, Germany).

### 4.7. Protein Carbonyl Assay (PCARB)

In order to measure protein carbonyls (PCARB), which serve as an indicator of protein oxidation, we utilized a dependable spectrophotometric assay that involved the use of 2,4-dinitrophenylhydrazine (DNPH) [53,54,55,58,59]. The formation of protein carbonyls (PCARB) can arise from the irreversible oxidation of particular amino acid side chains, including lysine, arginine, threonine, and proline, within the protein’s structure. Alternatively, PCARB may result from an augmented production of advanced glycation end products (AGE).

The human plasma samples were combined with a solution of 20% TCA and subjected to a 15 min incubation period on ice. Following the incubation period, the samples underwent centrifugation at a speed of 12,000 revolutions per minute for a duration of 5 min at a temperature of 4 °C, resulting in the separation of the supernatant. The pellets were subjected to treatment with a solution of 0.5 mL of 10 mM DNPH in 2.5 M hydrochloric acid (HCl). Next, they were incubated in a light-restricted environment for 60 min, with periodical shaking at 15 min intervals. Following the incubation period, a subsequent centrifugation step was conducted for a duration of 5 min at a speed of 12,000 revolutions per minute at a temperature of 4 °C. Subsequently, the pellet was treated with 10% trichloroacetic acid (TCA) and stored on ice for a period of 10 min. The samples were subjected to vortexing and subsequently separated through centrifugation. In order to eliminate surplus DNPH, two washing procedures were conducted employing a solution consisting of 1 mL of ethanol and ethyl acetate in a 1:1 volumetric ratio. The protein pellet was solubilized in a solution containing 1 mL of 5 M urea with a pH of 2.3. This solubilization process was carried out at a temperature of 37 °C for 10 min. The optical density was measured at a wavelength of 375 nm using an UV-VIS spectrophotometer (Kruss, Germany). The PCARB content was determined by utilizing the molar extinction coefficient of DNFH (22,000 M^−1^ cm^−1^). The concentration of PCARB was quantified and is reported in units of nanomoles per milligram of protein. The determination of the total protein concentration in the sample was conducted according the biuret method using a kit purchased from Diagnosticum Zrt. (Budapest, Hungary).

The other reagents used were provided by Sigma-Aldrich (St. Louis, MO, USA, DNPH, TCA) and Merck KGaA (Darmstadt, Germany) (solvents, urea, HCl).

### 4.8. Assay for Total Antioxidant Capacity (TAC)

The measurement of total antioxidant capacity (TAC) is commonly employed in the evaluation of the antioxidant status in human samples that are linked to various diseases. The TAC assessment demonstrates the overall ability of the body to combat oxidative stress through the production of antioxidant compounds. The assessment of this capacity in human plasma can be readily conducted through the utilization of a simple spectrophotometric method [53,54,55,56,60]. The samples were diluted at a ratio of 1:25 in a 1× phosphate-buffered saline (PBS) solution with a pH of 7.4. The diluted samples were then combined with a 0.1 mM 2,2 diphenyl-1-picrylhydrazyl (DPPH) reagent at a volume-to-volume ratio. The mixture was incubated in a dark environment for a duration of 30 min. Following the incubation period, the samples underwent separation through centrifugation for a duration of 3 min at a speed of 14,000 revolutions per minute. Subsequently, the optical density (OD) of the samples was measured at a wavelength of 520 nm utilizing a UV-VIS spectrophotometer. The expression of TAC was quantified in terms of millimoles of DPPH per liter (mmol DPPH/L).

DPPH was provided by Sigma-Aldrich (St. Louis, MO, USA) and the other reagents used were from Merck KGaA (Darmstadt, Germany).

### 4.9. Determination of Superoxide Dismutase (SOD) Activity

To assess the activity of superoxide dismutase (SOD), the blood samples underwent a procedure to induce hemolysis, as previously described [56]. Centrifugation was conducted at a force of 1100× *g* for a duration of 10 min to process a sample of 0.5 mL of blood. Subsequently, the upper layer of plasma was separated and extracted. The erythrocyte pellet was subjected to four washes using a physiologic saline solution. After each wash, centrifugation was performed for a duration of 10 min. Following the final wash, the erythrocytes were diluted with 2 mL of cold redistilled water, thoroughly mixed, and subsequently stored at a temperature of 4 °C for a period of 10 min. Prior to conducting the SOD assay using a Ransod kit (SD125, lot: 552218, Randox Laboratories, Crumlin, UK), it is imperative to perform a 100-fold dilution of the lysate with 0.01 M phosphate-buffered saline (PBS) at a pH of 7. Superoxide dismutase (SOD) activity was assessed by measuring the extent of inhibition in the reaction between superoxide radicals, produced through the oxidation of xanthine (0.05 mmol/L) by xanthine oxidase (80 UI/L), and 2-(4-iodophenyl)-3-(4-nitrophenol)-5-phenyltetrazolium chloride (INT, 0.025 mmol/L). The absorbance of the pink formazan was measured at 505 nm using a DU65 UV/VIS spectrophotometer (Beckman, Krefeld, Germany). A unit of superoxide dismutase (SOD) activity is defined as the amount of SOD required to achieve a 50% inhibition of the rate of INT reduction. To ascertain the degree of inhibition for each sample, the diluted samples and standard rates were transformed into percentages relative to the rate of the sample diluent, with the difference subtracted from 100%. These values were then assigned as the rates for the uninhibited reaction. The activity of superoxide dismutase (SOD) was quantified by measuring the amount of SOD units per milliliter of whole blood.

### 4.10. Glutathione Peroxidase (GPx) Assay

The measurement of GPx activity was conducted using the Ransel kit (lot: 564179/599552RS, Randox Laboratories, Crumlin, UK), as previously reported [56,61,62]. The enzyme GPx, derived from heparinized whole blood, facilitates the oxidation reaction between glutathione (GSH) at a concentration of 4 mmol/L and cumene hydroperoxide at a concentration of 0.18 mmol/L. The conversion of oxidized glutathione (GSSG) to reduced glutathione (GSH) is facilitated by the enzyme glutathione reductase, which has an activity level greater than 0.5 units per liter. This reaction occurs in the presence of NADPH, the reduced form of nicotinamide adenine dinucleotide phosphate, at a concentration of 0.34 millimoles per liter. The decrease in absorbance resulting from this reaction is subsequently measured at a wavelength of 340 nanometers. The measurement of optical density was conducted using a Beckman UV/VIS spectrophotometer (Kruss, Germany). In order to mitigate the potential interference of other blood peroxidases, a diluting agent and Drabkins’ reagent were used to dilute heparinized whole blood. The activity of GPx was quantified and is reported as units per liter (U/L) of hemolysate.

### 4.11. Reduced Glutathione (GSH) Assay

To assess the concentration of glutathione (GSH) activity, we combined equal amounts of blood and cold distilled water. The resulting mixture was then subjected to centrifugation at 4000× *g* for 15 min using an Eppendorf refrigerated centrifuge 5417 R (Eppendorf AG, Hamburg, Germany) to obtain a clear hemolysate.

Each hemolysate sample was subjected to treatment with 5% TCA, as previously reported [56]. The samples were then blended and separated through centrifugation at 4 °C for a duration of 5 min at a force of 28,000× *g*. Subsequently, the supernatants were pooled and mixed with a 0.01 M solution of 5,5′-dithio-bis-(2-nitrobenzoic acid) (DTNB, commonly known as Ellman’s reagent) in 0.07 M phosphate-buffered saline (PBS) at a ratio of 1:50 (*v*/*v*). The resulting mixture was then incubated for a duration of 45 min under dark conditions and at room temperature. The measurement of absorbance for the pale yellow product at a wavelength of 412 nm was conducted using a UV/VIS spectrophotometer manufactured by Kruss (Mainz, Germany). Subsequently, the obtained absorbance value was converted to glutathione (GSH) concentration using the GSH standard curve, and the concentration is expressed in milligrams per deciliter (mg/dL). DTNB and PBS were provided by Merck KGaA (Darmstadt, Germany).

### 4.12. Determination of Catalase (CAT) Activity

The Aebi method was employed to determine CAT activity, which involved the direct quantification of the rate of hydrogen peroxide decomposition by CAT and hemoglobin within the sample [56,63,64]. The samples underwent a dilution of 1:10 and were subsequently combined with 0.07 M PBS at a pH of 7. This was followed by an incubation period of 10 min at a temperature of 37 °C. The measurement of the decrease in absorbance at a wavelength of 240 nm following the addition of hydrogen peroxide was conducted using the DU65 UV/VIS Beckman spectrophotometer. The measurement of CAT activity was conducted by determining the molar extinction coefficient of hydrogen peroxide. In this context, one unit of CAT is defined as the amount required to decompose 1 mol of hydrogen peroxide within a duration of 1 min. Consequently, the activity of CAT was quantified as units per milligram of hemoglobin (U/mgHb) through the utilization of flow cytometry on automated analyzers. The reagents were provided by Merck KGaA (Darmstadt, Germany).

### 4.13. Neutrophil/Lymphocyte Ratio

The neutrophil and lymphocyte populations were quantified in peripheral blood samples collected through standard venipuncture using an automated flow cytometry analyzer (CELL-DYN Ruby System, Abbott Diagnostics, Wiesbaden, Germany). The calculation of the neutrophil/lymphocyte ratio (NLR) involved dividing the number of neutrophils by the number of lymphocytes [54,65].

### 4.14. Statistical Analyses

Statistical analyses were carried out using GraphPad software, version 10.1. The analysis employed an unpaired *t*-test for straightforward analysis, while a two-way ANOVA with Bonferroni correction was utilized to assess the relationship between the parameters and the severity of stroke as assessed by deficit scales. *p*-values that were equal to or less than 0.05 were considered to indicate statistical significance. The data are expressed as the mean value ± the standard error of the mean (SEM). Multiple linear regression was employed as part of the statistical analysis to examine the relationships and associations among the variables investigated in this study.

## 5. Conclusions

In conclusion, our study offers valuable insights on the intricate relationship between oxidative stress, antioxidant defense mechanisms, and inflammation in patients simultaneously afflicted by acute ischemic stroke and COVID-19 infection. The simultaneous increase in both markers of oxidative stress (TBARS) and antioxidant defense mechanisms (GPx, GSH, SOD, catalase) suggests a dynamic and adaptive response to oxidative stress in COVID-19 stroke patients. The exacerbated oxidative damage and heightened inflammatory response in the Stroke-COVID group underscore the need for tailored therapeutic approaches in this unique clinical scenario. Further investigations are warranted to elucidate the precise mechanisms driving these interactions and their clinical implications.

### Study Limitations

(1) This study might have a limited sample size, which can affect the generalizability of the results to a broader population. A larger and more diverse sample would have provided a more robust foundation for drawing conclusions. (2) Conducting the research at a single center may introduce bias and limit the representation of a more diverse patient population. (3) This study’s cross-sectional design allows for the observation of associations at a single point in time. Longitudinal studies would offer insights into how oxidative stress and antioxidant defense mechanisms evolve over time, providing a more comprehensive picture of the relationship. (4) This study may have selection bias if certain patients were more likely to be included in the study due to specific criteria. (5) Factors such as patients’ comorbidities, medications, and lifestyle choices may not have been fully considered.

## Figures and Tables

**Table 1:** Clinical and demographic data of the cohort.

Parameter	Control	Stroke-COVID	*p*-Value
Age–mean years (interval)	70 (42–87)	72.87 (59–89)	0.1493
Sex			
M (%) F (%)	61.1% 38.9%	42.5% 57.5%	0.0331 *
Comorbidities			
High blood pressure, N (%)	40 (86.9%)	48 (96%)	0.4171
Diabetes mellitus, N (%)	10 (21.7%)	13 (26%)	0.7530
Dyslipidaemia, N (%)	9 (19.5%)	28 (56%)	<0.0001 ****
Atrial fibrillation, N (%)	8 (17.3%)	15 (30%)	0.0659
Cigarette smoking			
No Yes	38 (82.60%) 8 (10.86%)	43 (86%) 7 (6%)	0.9254
Stroke evaluation			
Right hemisphere, N (%) Left hemisphere, N (%)	20 (43.47%) 26 (56.52%)	24 (48%) 26 (52%)	0.6609
NIHSS evaluation			
5–15 (Moderate), N (%) 16–20 (Moderate to severe), N (%)	31 (67.39%) 15 (32.60%)	38 (76%) 12 (24%)	0.3539
COVID-19 Severity Scale			
Scores 1, 2 or 3 (Mild) Scores 4 or 5 (Moderate) Scores 6, 7, 8 or 9 (Severe)	N/A	17 (34%) 30 (60%) 3 (6%)	

* *p* < 0.05; **** *p* < 0.0001; N/A: Not applicable.

**Table 2:** Univariate and multivariate analysis of oxidative stress and inflammatory response markers.

Parameter	Univariate Analysis 95% CI	*p*-Value	Multivariate Analysis 95% CI	*p*-Value
Oxidative damage markers				
TBARS	0.4948 to 0.6735	<0.0001 ****	0.5780 to 0.8762	<0.0001 ****
PCARB	−0.0975 to 1.602	0.0823	−0.0399 to −0.0026	0.0259 *
Antioxidant defense markers				
TAC	−2.350 to 3.982	0.6114	−0.0076 to 0.0037	0.4988
GPx	54.36 to 147.3	<0.0001 ****	0.0004 to 0.0011	<0.0001 ****
SOD	2.150 to 64.43	0.0363 *	−0.0006 to 0.0004	0.7183
GSH	0.1256 to 1.092	0.0139 *	−0.0196 to 0.0522	0.3708
CAT	147.3 to 2025	0.0237 *	−3.227 × 10^−5^ to 3.911 × 10^−6^	0.1233
Inflammatory response				
NLR	0.1441 to 1.750	0.0211 *	0.0036 to 0.0463	0.0220 *
CRP	57.14 to 126.2	<0.0001 ****	0.0004 to 0.0015	0.0008 ***
ESR	1.166 to 20.95	0.0287 *	−0.0003 to 0.0028	0.1170

* *p* < 0.05; *** *p* < 0.001; **** *p* < 0.0001.

**Table 3:** Multiple linear regression of NIHSS with oxidative stress, antioxidant defense, and inflammation markers.

Parameter	Estimate	Multivariate Analysis 95% CI	*p*-Value
Oxidative damage markers			
TBARS PCARB	2.259 −0.05305	0.2875 to 4.231 −0.2448 to 0.1387	0.0251 * 0.5847
Antioxidant defense markers			
TAC	−0.08283	−0.1386 to −0.02706	0.0039 **
GPx	0.001360	−0.002367 to 0.005088	0.4713
SOD	−0.004031	−0.009804 to 0.001742	0.1693
GSH	0.0006443	−0.3625 to 0.3638	0.9972
CAT	2.833 × 10^−5^	−0.00015 to 0.00021	0.7607
Inflammatory response			
NLR	1.586	−0.3910 to 0.0433	0.1156
CRP	2.000	5.675 × 10^−5^ to 0.01161	0.0478 *
ESR	0.7031	−0.01048 to 0.02202	0.4834
Rankin scale	4.983	0.9630 to 2.233	<0.0001 ****
MRC scale	2.861	−1.751 to −0.3184	0.0050 **

* *p* < 0.05; ** *p* < 0.01; **** *p* < 0.0001.

## Data Availability

Data are contained within the article.

## Supplementary Materials

The supporting information can be downloaded at: https://www.mdpi.com/article/10.3390/ijms242316790/s1.
