# Supplementary material for: Oxidative Stress and Antioxidant Defense Mechanisms in Acute Ischemic Stroke Patients with Concurrent COVID-19 Infection
Source: Int J Mol Sci. 2023 Nov 27;24(23):16790. doi: 10.3390/ijms242316790 (PMC10706234; doi:10.3390/ijms242316790)
Supplement: Supplementary file 1 [file ijms-24-16790-s001.zip › ijms-2703600-supplementary.pdf]

Supplementary Table S1 – Modified Rankin Scale Score.

Modified Rankin Scale	
0	No symptoms
1	No significant disability. Able to carry out all usual activities, despite some symptoms
2	Slight disability. Able to look after own affairs without assistance, but unable to carry out all previous activities
3	Moderate disability. Requires some help, but able to walk unassisted
4	Moderately severe disability. Unable to attend to own bodily needs without assistance, and unable to walk unassisted
5	Severe disability. Requires constant nursing care and attention, bedridden, incontinent
6	Dead

Supplementary Table S2 – Medical Research Council (MRC) muscle strength scale Score.

Medical Research Council (MRC) muscle strength scale	
0	No muscle contraction or movement observed
1	Muscle contraction is present, but there is no joint movement
2	The muscle can move the joint when gravity is eliminated (passive movement)
3	The muscle can move the joint against gravity, but not against any added resistance
4	The muscle can move the joint against some resistance but is weaker than normal
5	Normal muscle strength, with the joint able to move against full resistance

Supplementary Table S3 – The National Institutes of Health Stroke Scale (NIHSS).

**The National Institutes of Health Stroke Scale (NIHSS)**		
Level of consciousness	0	Alert
	1	Not alert, arousable
	2	Not alert, obtunded
	3	Unresponsive
LOC questions	0	Answers both correctly
	1	Answers one correctly
	2	Incorrect
LOC commands	0	Obeys both correctly
	1	Obeys one correctly
	2	Incorrect
Gaze	0	Normal
	1	Partial gaze palsy
	2	Forced deviation
Visual fields	0	No visual loss
	1	Partial hemianopsia
	2	Complete hemianopsia
	3	Bilateral hemianopsia
Facial palsy	0	Normal
	1	Minor paralysis
	2	Partial paralysis
	3	Complete paralysis
Motor arm (A)Left; (B) Right	0	No drift
	1	Drift before 10s
	2	Falls before 10s
	3	No effort against gravity
	4	No movement
Motor leg (A)Left; (B) Right	0	No drift
	1	Drift before 10s
	2	Falls before 10s

	3	No effort against gravity
	4	No movement
Ataxia	0	Absent
	1	One limb
	2	Two limbs
Sensory	0	Normal
	1	Mild loss
	2	Severe loss
Language	0	Normal
	1	Mild aphasia
	2	Severe aphasia
	3	Mute or global aphasia
Dysarthria	0	Normal
	1	Mild
	2	Severe
Extinction/inattention	0	Normal
	1	Mild
	2	Severe
